# A rare syndrome of central diabetes insipidus with spastic cerebellar ataxia

**DOI:** 10.4103/0972-2327.40226

**Published:** 2008

**Authors:** Subramanian Kannan, Sivaprakash, G. Usha, V. Raji

**Affiliations:** Institute of Internal Medicine, Madras Medical College, Government General Hospital, Chennai - 600 001, India

**Keywords:** Central diabetes insipidus, normal posterior pituitary bright spot, spastic cerebellar ataxia

## Abstract

The syndrome of central diabetes insipidus (cDI) and spastic cerebellar ataxia is rare with only a few reports in the literature. We report the case of a 21-year-old patient who was diagnosed to have central diabetes insipidus at the age of 7 years and presented to us at the age of 21 years with progressive spastic cerebellar ataxia that evolved over four years. His MRI showed normal hyperintense signal from the posterior pituitary. The persistence of posterior pituitary signal in patients with cDI is unusual and is observed in the familial variety of cDI, the possible etiology in our patient. A brief review of the literature on the rare syndromic association of cerebellar ataxia and cDI has been discussed.

## Introduction

Central diabetes insipidus (cDI) is a heterogeneous condition characterized by polyuria and polydipsia due to arginine vasopressin (AVP) deficiency. In 30-50% cases, it is idiopathic.[1] In the rest of the cases, it may be due to hypothalamic-pituitary stalk lesions such as Langerhans cell histiocytosis (LCH), idiopathic granulomatosis or neoplastic process. Rarely, diabetes insipidus is familial with autosomal dominant inheritance.

## Case History

This case presentation is rare with four cases previously reported in the literature.

A 21-year-old male, born of nonconsanguineous parentage presented with intentional tremors and asymmetrical weakness of limbs (left > right). The symptomatology had an insidious onset and was slowly progressive for 6 years. There was no history of behavioral changes, memory disturbance, sensory or extrapyramidal symptoms. He was diagnosed to have central diabetes insipidus (cDI) at the age of 7 years and had been on oral vasopressin 100 mcg twice a day. There was no family history of similar disorders. Rest of the physical examination was within normal limits. Neurological examination disclosed normal cognitive function with ataxic dysarthria. Cranial nerves examination revealed gaze-evoked nystagmus and slow saccades. On motor system examination, there was increased tone in all the four limbs and power was 4/5 on the right and 3/5 on the left. There was impaired coordination in the upper and lower limbs. Gait was ataxic with exaggerated deep tendon reflexes and bilateral extensor plantar response. Rest of the examination was normal.

**Figure 1:**
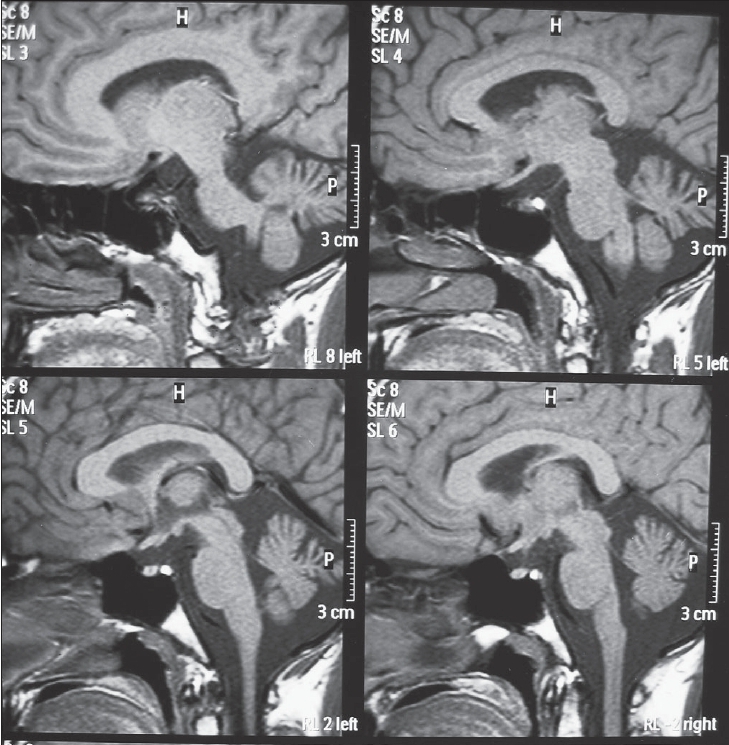
Atrophic cerebellum and normal posterior pituitary bright spot

Basic biochemical and blood studies were normal. The MRI brain (T1W) showed normal signal from posterior lobe of pituitary gland with small infundibulum and diffuse cerebellar and brainstem atrophy [Figure 1]. The endocrine evaluation for anterior pituitary hormones was normal. Skeletal survey including a Tc bone scan was normal.

## Discussion

The posterior pituitary is seen as a hyper-intense bright spot in the T1W images in the MRI of normal persons. The appearance of the posterior pituitary signal has been ascribed to fat within the sella, lipid accumulation within the pituicytes or the secretory granules containing AVP. In fact, the hyperintensity of the posterior pituitary usually accompanies hypothalamic-posterior pituitary functional integrity. The clinical and biochemical diagnosis of diabetes insipidus is supported by the absence of the posterior pituitary bright signal on MR T1W images and may be an early evidence of hypothalamic-pituitary lesion.[1] The persistent hyperintensity of the posterior pituitary has been described primarily in patients with familial autosomal dominant central diabetes insipidus and rarely in patients with idiopathic or lesional central diabetes insipidus.[1] In our patient, there is persistence of posterior pituitary signal, despite the presence of central DI for 14 years that supports a familial etiology for the cDI. Familial cDI may be autosomal dominant or autosomal recessive; however, the absence of family history does not exclude this possibility.

The distinctive combination of cranial DI and spastic cerebellar ataxia is rare and has been previously reported.[2–4] Langerhans cell histiocytosis (LCH), Erdheim-Chester disease (ECD), calcifications, tumors, multifocal eosinophilic granuloma and degenerative changes are the causes for this syndrome complex of cDI and cerebellar ataxia. In LCH, this occurs due to either an infiltrative process or as a paraneoplastic phenomenon. Erdheim-Chester Disease is a non-LCH histiocytic infiltration of different organs and bones and can present with a combination of cDI and ataxia. In such cases, MRI reveals intra-axial hyperintense lesions on T2W with gadolinium enhancement. Posterior pituitary signal may be absent or retained with enlargement of infundibulum.[4]

Hence, a detailed neurological examination and investigation is needed in patients with cDI who present later with neurological symptoms.
